# Artificial Intelligence-Based Robotic Technique for Reusable Waste Materials

**DOI:** 10.1155/2022/2073482

**Published:** 2022-05-06

**Authors:** Pravin R. Kshirsagar, Neeraj Kumar, Ahmed H. Almulihi, Fawaz Alassery, Asif Irshad Khan, Saiful Islam, Jyoti P. Rothe, D. B. V. Jagannadham, Kenenisa Dekeba

**Affiliations:** ^**1**^ Department of Artificial Intelligence, G. H. Raisoni College of Engineering, Nagpur 440016, India; ^2^Department of Computer Science &Engineering, Chitkara University, Chandigarh, Punjab, India; ^3^Department of Computer Engineering, College of Computers and Information Technology, Taif University, P.O. Box 11099, Taif 21944, Saudi Arabia; ^4^Department of Computer Science, Faculty of Computing and Information Technology, King Abdulaziz University, Jeddah 21589, Saudi Arabia; ^5^Civil Engineering Department, College of Engineering, King Khalid University, Abha 61411, Asir, Saudi Arabia; ^6^Department of Electrical Engineering, St. Vincent Pallotti College of Engineering & Technology, Nagpur, India; ^7^Department of Electronics & Communication Engineering, Gayatri Vidya Parishad College of Engineering(A), Madhurawada, Visakhapatnam-530041, India; ^8^Department of Food Process Engineering, College of Engineering and Technology, Wolkite University, Wolkite, Ethiopia

## Abstract

Waste management is a critical problem for every country, whether it is developed or developing. Selecting and managing waste are a critical part of preserving the environment and maximizing resource efficiency. In addition to reducing trash and disposal, reusable items are predicted to be of great benefit since they lessen our dependence on raw materials. The usage of compostable trash may be expanded outside fertilizers and dung after the metallic, chemicals, and glass items have been recycled. After a good scrubbing, the glass may be broken down and remelted to create new items. Reusing waste items via garbage recovery is one of the best methods to do so. This document outlines the steps that must be taken to maximize the use of garbage. This work describes a reusable industrial robot arm for grasping and sorting things depending on the resources they contain. Gripping, motion control, and object material categorization are all integrated into a full-automation, reusable system architecture in this study. LeNet also was adjusted to classify garbage into cartons and plastics using an artificial intelligent technique, with the use of a customized LeNet model. Movement in terms of moving the robot in the most efficient way possible, the robot's grabbing, and categorization were incorporated into the movement design process. The system's grabbing and object categorization success rates and computation time are calculated as metrics for evaluation.

## 1. Introduction

In pollution prevention, wastes and the accompanying dangers have become a growing concern. Increasing attention is being paid to waste management across the globe, including both the emergence of future waste-reducing techniques and those connected to the disposal and monetary value of waste products. Unreasonable approaches to material procurement are a primary cause of excessive waste production. Waste that accumulates in dumps may be exploited to make recycling of materials resources worth a few 100 million dollars. Coal accounts for 25 percent; copper, lead, steel, or other materials make up 35 percent; and ash, sludge, rock refuse, aggregates, and other constituents make up 40 percent [1]. No amount of social and technical synchronization can keep the amount of trash created in a given location at a level that determines the relationship amongst raw materials, environmental waste, and medical garbage.

The application of current low-waste or non-waste technology, the reuse of raw resources, and the replacement of historically used natural resources should all be considered in efforts to reduce waste. [2] Low and waste of time innovations are the ideal solution to the issue of industrial waste contaminating the surrounding ecosystems. The foundation of non-waste technology (NWT) is waste prevention and complete input material use. With the help of several technical methods, pollution may be completely eliminated without causing any negative impact on our ecosystem. Waste must not be put in this situation [3]. id Nwt was enabled, and then the waste prevention of foreign raw materials will be reduced. Lowering the amount of energy used to handle garbage is another way to cut down on power, heat, or technological use. Additionally, the use of non-waste equipment reduces material use, atmospheric losses, and operational expenses [4]. Recycling may also help to cut down on trash. When it comes to its primary function, it optimizes the reuse of resources and lowers the amount spent on treatment.

Recycling occurs in two ways: first, in the manufacture of things, and second, in the development of garbage from those commodities [5]. It is predicated on the notion that makers would adopt attitudes that promote the development of the most recovering materials and that receivers will adopt behaviours that reflect these views. For example, recycled waste trash may be recycled by reusing raw materials and changing their quality and structure, amongst other methods [6]. Sorting garbage not just into metallic, bio, plastics, newspaper, or glass sectors is required for this. Nowadays not all materials are acceptable for disposal, and it is required to employ sophisticated procedures to identify the kind of component in each category.

Today, robots are used in a broad range of industries. An entirely new way of doing business known as “Economy 4.0” is taking shape because of the fast development of industrial robots. The ultimate objective of Industrial Revolution 4.0 is to increase efficiency while increasing automation. Different engineering robotic technologies have been explored, including smart city commuting and manufacturing [7]. Some important techniques must be improved to develop an urban environment in order to increase the effectiveness of life for humans. For example, an automated garbage recycling system might enhance human well-being while also safeguarding the planet.

Item identification, object categorization, and actuation are all necessary components of an autonomous trash reusing system. Furthermore, the development of the system necessitates a sophisticated manipulating scenario. These features must be interconnected and discussed with one another to build a reusable framework [8]. In a manufacturing environment, employees' interactions with the robotic platform are also taken into account. An autonomous recycling method was devised in this study. A multipurpose gripping tool, trajectory tracking, object categorization, and interconnection of all parts were the four key issue areas that were addressed in the development of the system [9]. Trying to follow is one of the journal's achievements.

A redesigned LeNet model is used to categorize waste products using machine learning. Plastic and cartons are the two primary kinds of things that may be categorized using this methodology. The experimental set of statistics such as rotating and lighting was used to enhance the model once it had been built [10]. In the first collection of data, using an efficient flow of information, we were able to construct paths that were best suited to the robot's arm motions and create a suitable grabbing stance. The placement of recyclable trash products in a specified region was also made easier with the use of motion control. A novel paradigm for an automatically reusable organization involves features for perceiving, manipulating, and classifying objects.

## 2. Objective

According to this publication's principal goal, waste material may be reused effectively. To assure the proper utilization of waste material, several measurement operations need to be carried out. In the suggested system, multipurpose grabbing is used to construct an automatically reusable framework that otherwise chooses the things that classifies through AI technology.

## 3. Review of Literature

According to Gundupalli et al. [6], a thorough market investigation was conducted to identify the various economic options available to include controlling the electromechanical waste treatment procedure [11]. An automaton garbage sorting equipment incorporating AI technology, known as the ZRR2 technology, is one of the possibilities being investigated (AI). Construction and demolition (C&D) wastes may be separated by any of these machines, which are outfitted with object recognition and learning algorithms. Four distinct fractions ranging up to 98 percent purity may be handled by one ZRR robot arm. Aside from building and deconstruction waste, ZRR robots are only now being used in this area because of their efficiency and lower cost of waste segregation for materials such as brick.

According to Ni et al. [8], specialization is the process of splitting a satellite image into many homogenous sections, which is useful for studying the picture in a variety of ways, such as identifying and detecting objects, categorization, and principal component analysis; this approach is called segmented. To quickly and accurately segment lowland and aeronautical LiDAR point dumps, we suggested a reticuloendothelial approach for producing countries.

According to Karningsih et al.[9], in a development or a development, dependability, shortfalls, and numerous delays are perhaps the most common issues. As a result, a lean type of methodology has been advocated to remove these worries from building procedures, which is very economical for construction. According to the authors' comprehensive testing, the greatest amount of waste is caused by rectification and awaiting. These sources of waste may be minimized using this method. The main problem of this strategy is that it has not been thoroughly evaluated to see how effective it is.

According to Kshirsagar et al. [4], numerous modeling techniques, with one-layer inhibitory, first-layered assumptions, RBF, FFNN, PNNN, GRNN, etc., were designed to fulfil its goals. For a restricted set of modestly implemented knowledge and designs, this unique human brain was thought to be optimal. However, the amount of data that can be processed and the amount of time it takes to construct solutions have been constrained by new technical developments. Traditional machine learning was then altered, permitting for a far larger number of recommendations and a much shorter period to collect. Complex and dependable information results may be described or delivered using machine learning.

According to Covaciu et al. [12], this article shows how vast amounts of rubbish and waste materials are generated by demolishing and building operations. As the population continues to rise, more and more homes are being constructed throughout the globe to support them [3]. The scientists of this work have sought to limit this output by reusing the plastic waste for a variety of building projects. A low-energy electromagnetic field has been used by the authors to determine the garbage and its qualities to efficiently repurpose the waste. This publication's main issue is that recycling can only minimize a modest amount of litter.

Masuduzzaman [11] provides a solution to these difficulties and a considerable rise in electromechanical waste. Technological trash must be regulated since it contaminates the ecosystem and may be dangerous to people and animals in the vicinity of pollution. Because of this, the writers have decided to use electromagnetic wastes as an admixture in concrete to address this issue [5]. Electronic garbage, according to the scientists, may drastically cut waste creation if utilized in aggregation. The main problem of this strategy is that the developers have not evaluated these strategies for different metrics.

Savoshinsky et al. [13]emphasize that the local authority is essential for the protection of its communities and minimizing the destruction caused by fires. Many building materials may mix with each other to form a lethal poison that is highly combustible, and this is a primary cause of fires. For this reason, trash management is necessary for the sustainability of waste disposal around the municipality. [8] Hence, waste management uses GPS for administration not for prevention, and, this system has a severe disadvantage.

## 4. Reusable Waste Materials

It is shocking to see how much plastic is being produced on a worldwide basis. More polyethylene has been manufactured in the past decade than that in the preceding century combined [7]. This so-called plastic material movement, in which scientists explore novel techniques to test the envelope of polymers, is responsible for this overall increase in plastic product manufacturing [8]. Plastic goods such as microplastics have been targeted by new environmental regulations [9]. In the recent two decades, a number of comprehensive literature evaluations have been written to showcase the currently regarded country disposable plastic technologies. Different initiatives for reusing plastic, which include incorporating reusable materials with virgin polycarbonate and using stabilizers, experiments on the copolymerization of varying plastic products, and reinforcing polymer composites with plant fabrics or carbon fibres to begin producing lightweight structures with high mechanical strength, are presented in these recommendations.

There is an increasing number of people in the industrialized world calling for significant shift away from single-use plastics such as food and household packaging in favor of reusable alternatives. Collecting low population density, elastomeric, and thermoplastics is the sole common methodology for home waste plastic [14], and typically, the recovered material must be handled in ways that limit contamination [15].

**Figure 1:**
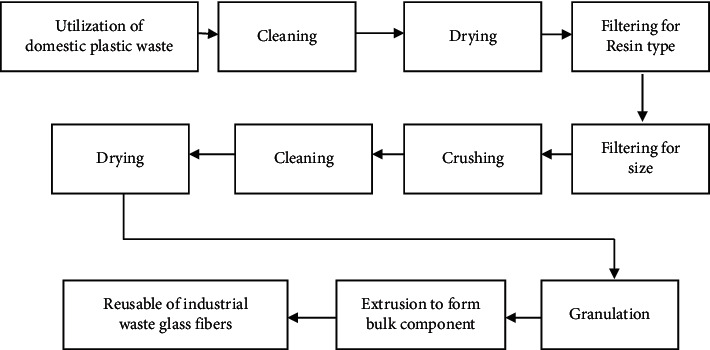
Traditional reusable route.

As shown in Figure 1, current recyclable processes include screening for size and shape, extensive cleaning and separating, crushing, dryness, and agglomeration to generate a reusable recovered resin powder commodity [14]. Poor plastic reuse rates are a result of the complex screening procedure and the low monetary value of plastic wastes [16]. Reclaimed polymeric, in comparison with virgin polymers, may degrade in qualities more easily than virgin polymer matrix because of the overheating, contaminating, and group members of polymer blend grades [15]. A product generated by separating various polymers into separate phases has a limited range of applications. By artificial intelligence model for Parkinson disease prediction using healthcare sensor sorting, separating plastics before crushed, and shredding, phase segregation can be eliminated. Nonfood container, traffic concrete blocks and signs, lawn furniture, and laminated veneer are just a few examples of the very tiny fraction of thermoplastics made from recycled materials now in use.

## 5. System Architecture

**Figure 2:**
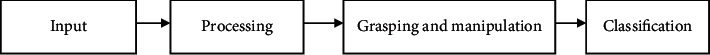
System architecture.

(a) Feature extraction technique, (b) gripping specialist team, (c) product component categorization, and (d) optimization algorithm are all components of this method. Kinect captures a satellite image of data, which is then processed to produce groupings that describe each item within the box. Then, the manipulator plots the best route to each item and selects the appropriate gripping approach based on the object's size. A redesigned LeNet algorithm is programmed to find the substance of an item after the grabbing process [12]. The RGB camera is also used to extract the properties of the items that are best suited for classification amongst cartons and polyester. Outside of the building, an arm moves the thing to a shipping box after determining its substance.  Figure 2 depicts the usage of two separate distribution boxes: one for cartons and one for plastics.

### 5.1. Object Segmentation

There are various state-of-the-art methods in the Polygon Mesh Libraries, encompassing component estimating, developing capabilities, and the model is shown in Figure 2, and recognition, which are all free source. The photogrammetric data are analyzed to derive the forms of the recyclable materials [17]. It is separated into two main stages of image recognition: the first step is to collect the data in the form of photogrammetry, and the second is to filter the workstation. The third step is to cluster individual items into groups, and the fourth step is to create a plane modeling and determine the plane's fundamental. The Kinect was used to collect cloud data, which were then fixed to a foundation and connected to the building [18]. Throw filtering was installed all along phone camera axis once the data collecting process was completed.

### 5.2. Confirmatory Factor Analyses for Object Manipulating

Dependable grasping activities without preset estimate of grasping stance are the system's aim. For this purpose, a versatile resultant force was developed that can be used with both pressure and grasping tools. A resultant force with an extendable manner analogous to this was also utilized to recognize and grab things and switch amongst pressure and grasping modes automatically [11]. To choose the best tool for grabbing an item, PCA was utilized. To determine whether pressure or gripping is better, we measure the items' measurements and compared them to the gripper's openness. If the products' measurements will be more than the gripper's introduction, we utilize suction.

### 5.3. Classification System

Once trash items have already been collected up and placed beside an RGB camera, the suggested identification system's purpose is to sort them into two distinct groups [19]. To increase generalization ability, we have decided to take the methodology of identifying and categorizing each item independently.

### 5.4. Motion Planning for Grasping Objects

**Figure 3:**
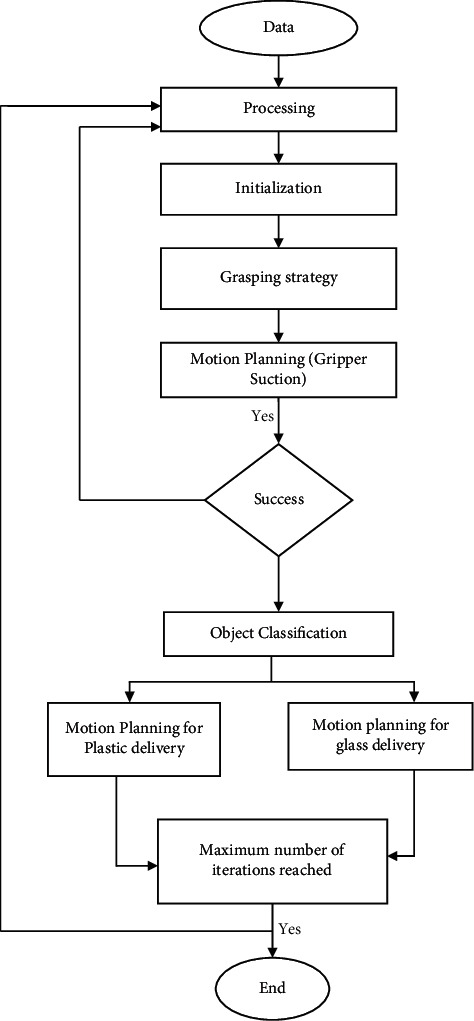
Flow chart of the robotic reusable system.

Motion control is often used for decades to find the best way to maneuver a robot. KDL and OMPL, in particular, are often employed to search for motions of a robotics arm's mechanics. Move it! Figure 3 was implemented into ROS as a component to provide personality avoiding using Laplace transform to assess the viability of grab in the system design. In addition, Move it sampling-based !'s planning feature may suggest a number of alternative routes to the desired destination [13].

URDF (the ROS standardized robot specification format) collision regions were set up before utilizing Move it! to prevent collisions here between robotic manipulator and objects. Additionally, the relational robot characterization format established particular locations, including fulfillment centers, the beginning orientation of the robot arm, and SRDF. Throughout trajectory tracking, it was permitted to employ the process to improve method since it aids in the search for a more ideal route than the prior one. To provide a barrier and maintain a steady end effector attitude, we employed a trajectory monitoring approach, which generated compass bearings between some of the arm and target.

## 6. Methodology

### 6.1. Architecture of Convolution Neural Network

**Figure 4:**
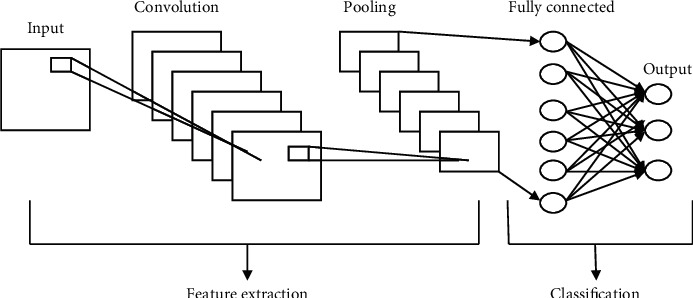
Architecture of CNN.

CNNs are perhaps the most frequently used photograph systems. CNNs have an impact on the visual field of the observer. Several imaging methods, such as feature and scene understanding, are at the cutting edge [16]. Distinct information approaches differ greatly in the way CNNs combine feature extraction methods. As shown in Figure 4, a basic CNN pattern may be observed. Intake, convolutional, pooling, fully connected layers, as well as output neurons, make up the system's many layers.

Extraction and categorization of characteristics are the 2 types that all these layers fall under. A convolution, a bundling layer, and a totally coupled output units are all that are needed to recover the capability. If the input photographs need to be downsized, the input layer establishes a constant value for them. A fully connected layer with numerous learned kernels is then used to equalize the image and to keep information in the picture intact of polling layer size [20]. The results of functionality extraction are classified as ideation. In the grouping, each layer's properties are brought together. Lastly, there is one output neuron for each kind of object in the output layer. The marking outcome is the conclusion of the process of assessment.

The waste management input is where the weighing sensor is plugged in to measure the garbage's mass. The price of each particular byproduct is immediately computed based on its mass. Nonorganic, recyclable objects are also shown on the screen through a currently residing monitor. Identification and monitoring of targets were accomplished using alexnet model. It is first possible to investigate the topic, then segregate it, and ultimately develop a model that appropriately identifies the subject.

Numerical techniques may be used to identify items. However, to increase the network's efficiency, a large study of data is required. Over 15 million pictures classified with categories [10] are used in AlexNet, an instructional model. The AlexNet framework has already been constructed with 25 layers to taking use of this massive data set. Even before algorithm was updated and altered to detect ten distinct things, this is what it looked like.

As an example, consider an image representation in *Fi* (*i* = 1, 2,…, where the total number of character mappings is shown). The weighted measurements of a spreading patch are related to other operation layer mappings in a similar way. There will be an infinite number different weight metrics if the other functional map (*Oj* (*j* = 1, 2,…, *J*)) is specified as *Oj* (*j* = 1, 2,…, *J*). The weights of *wi*,*j*=(*i* = 1,2,…, *I*; *j* = 1,2,…., *j*) connections are taken into consideration.

In data processing, mappings may be illustrated by employing the transformation function. This continuity formula may be used to assess each functionality mapping unit there in convolution: (1)

(1) $$\begin{array}{c} q_{j,m}=\sigma \left(\overset{I}{\underset{i=1}{\sum }}\sum_{n=1}^{f}F_{i,n+m1}W_{i,j,n}+W_{F,j}\right). \end{array}$$

When calculating the optimal computational model for covariances, the accompanying calculation [1] shows how fully connected capability has been used:

(2) $$\begin{array}{c} p_{i,m}=c\sum_{n=1}^{H}q_{i\left(m-1\right)\times x+1}. \end{array}$$

The scaling parameter is denoted by *c* in this case. An activating function is designed on the evolutionary algorithm. Here, you will find CNN's most extensive level. As a means of reducing the likelihood of an improper fit, the fall layers were developed.

Once trash items have already been gathered up and placed before an RGB camera, the suggested categorization system's purpose is to sort them into two distinct groups. To increase classification performance, we have decided to take the technique of identifying and categorizing each item independently [17]. This had been based on the development of a customized LeNet 5 modeling, which is able to recognize characters from RGB pictures of 150 × 150 pixels, which are much larger than the ones typically utilized for character segmentation by conventional LeNet models. In the first fully connected layers, there are 32 filters, and in the final convolution operation, there are 64 filters, each of which is 3 × 3. The linear transfer function is accompanied by a 2 × 2 highest speed both in the *x* and *y* directions with a stride of 1 for each convolution layer. To prevent overfitting, regularization was done during the first 64-unit dense layer, with a washout term of 0. Finally, there is a thick layer of 2 units, which denotes the number of class labels used for trash categorization. Using a dataset of normalized RGB photographs of garbage items with adjacent pixels scaled from zero to 1, the suggested classifier was tested. A machine learning technique with a computed value of 0.01 was used in the optimization procedure.

### 6.2. Hardware System Description

During the CENTAURO International Program-iSort, a robotics recycle system was created using the suggested robotic framework (2016–2018). You will find the robot arm (UR5), distance camera (Microsoft Kinect V2), two adhesive cups (small and huge), and Inspiron mono-camera in the enclosure. Capabilities that detect trash things and categorize them depending on the product may be implemented in this system. As though it were being built in a manufacturing environment, the experimental procedure was evaluated. However, owing to safety concerns and limited space, we were unable to erect a similar structure. Since the robotic and person were protected, the agency's pace was also slowed.

### 6.3. Collecting the Dataset

A camera positioned at various angles gathered data on glasses and polymers before it was used to take photographs for the training portion. With the help of ROS bag, points of origin were obtained directly. A total of 103 sample photographs, 50 of which would be glassware and 53 of which would be plastics, were obtained. However, the dataset was too little to fully use the capabilities of the CNNs. Image enhancement was done to the training sample using a number of random changes in overcoming the restriction of training sample. Consequently, the dataset had a greater variety of training photographs and never saw the same images again. As a result of this strategy, the CNN architecture is able to generalize to a wide range of real-world scenarios.

### 6.4. System Initialization

It was necessary to get out from the way of the work area so that the depth camera system could see the robot arm attached to the steel plate in its entirety. The workplace was positioned roughly 40 cm below the surface of a pedestal with four steel bars because it was thought to be the easiest spot to grip items. A RGB camera was put in the front of the robot arm's starting location for object categorization. Two boxes had been set aside for the collection of unwanted items first from arm's consignment. During the functioning of the recycling system, the starting locations of the arms, cameras, and compartments for the garbage were all predetermined. Thirty glassware and polymer items were constructed for the research, which included the testing: grabbing an item that used a multipurpose grabbing tool (siphon tube and gripping) and classifying object materials. As a result of this, if one carton was analyzed with two distinct item combinations, it was classified as two separate instances. Furthermore, if a gripping tool was unable to take up an item instinctively, the thing was eliminated by the specified tool.

## 7. Result and Discussion

### 7.1. Success Rate

When grabbing with pressure or a grabber, the results are shown in Tables 1 and 2. These tables are subdivided into categories depending on the spatial and material features of the items.

Employing pressure, 13 voluminous glass (VG) and 10 non-voluminous glass (NVG) items were evaluated for gripping carton, and each item was examined 5 times. In the plastics items, four VP and six NVP samples were analyzed in the same way. When it comes to sucking, plastic (91 percent success rate) beats glass (79 percent) (87.12 percent). Gripping a tiny piece of glass was a major challenge since the pressure cannot really reach its grabbing point since its fragmentation could not identify it. Suction collapsed when the item was too high since the tool was under that much stress. Many other concerns arose owing to mechanical problems, the existence of holes and many other imperfections on item surfaces, and the inclusion of adhesive tape in a few of the carton examples. Caused by plastic pattern's elastic interface and minor plastic breaking following extraction, segment issues were encountered. Using a grabber, the the glass samples are better than ployster sampling (87.78 percent) in this area (69.86 percent). The fundamental challenge with gripping is that there is a limited amount of space.

**Figure 6:**
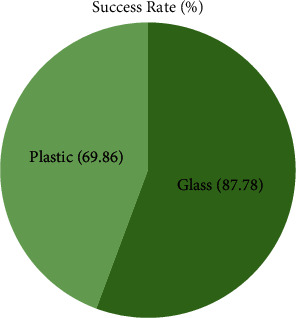
Success rate of grasping objects using gripper.

In Figure 6 as a result, physical constraints on rotating joints thwarted the robot and/or caused the manipulation to strike the item. The thickness of a sample and the incorrect fragmentation of an item constituted two additional minor problems. As a result of the experiments, it was determined that pressure was the most effective method for removing plastic samples, whereas gripping them was the least effective one.

Table 3 illustrates the outcomes of categorization trials in relation to the classification portion. Although the rate of success for glass and metal samples is comparable, plastic sampling has such a somewhat better success rate (87.1 percent) than prepared samples (86.4 percent). As with the trials involving gripping, these two percent are relatively high. The samples' colors were to blame for the difficulties encountered during categorization testing. These containers were mistaken for plastic because of their closeness to the plastic hues. As a result, the plastic was mistakenly classified as a glass, even though it had not been included in the entire data. The absence of sunshine also hampered the categorization of the packing, as it did for the samples.

### 7.2. Accuracy and Loss of Train and Test Phase Results

**Figure 7:**
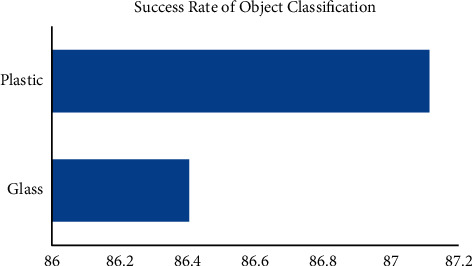
Success rate of object classifications.

**Figure 8:**
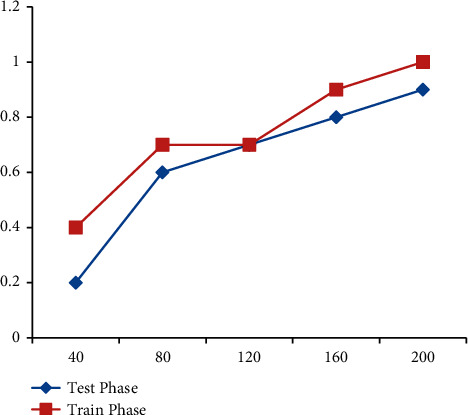
Accuracy and loss of train and test phase results.

In Figures 7 and 8, the efficiency on both training and test sets was measured across 260 cycles. The network's performance in testing and training is shown in blue and red, correspondingly. In Table 4, after training, efficiency achieves a value of 0.99, and in the test phase, it is 0.96. In the training phase, the optimizing method leads to a loss function of 0.01, and in the test phase, it leads to a loss function of 0.08. Overfitting does not have an effect on the model since it can operate and generalize well with new information.

## 8. Conclusion

**A**n autonomous robot platform was shown in this study. Reusable practices in enterprises may be encouraged by this system's ability to grab and categorize things based on their composites (glass or plastic). An industrialized sorted management system and distinctive robotic architecture with photo-editing, motion control, grabbing, and classifications are two of the primary innovations in this study. Additionally, the employment of a multimodal end effector furnished including both gripping and pressure tools boosted the chances of success even during grabbing procedure, hence minimizing the risk of mistake during this job. Only the larger suction was employed throughout the testing. Both forms of pressure are used to determine which one is best for the task at hand. We did not take into account grasping in crowded situations as a constraint of the proposed approach. As a result, this gripping area may be a difficult one to study in the future. For the grading system to generalize more easily to new items, the entire data must be combined to create a large-scale data. Additionally, a novel strategy that relies on learning additional characteristics should really be discovered to improve the categorization of substances. Ultimately, addressing a wider range of items and different types of plastic might be a tough notion that would generalize a highly autonomous reusable technology. In future, deep learning model is combinedly used for the waste material management.

## Figures and Tables

**Figure 5:**
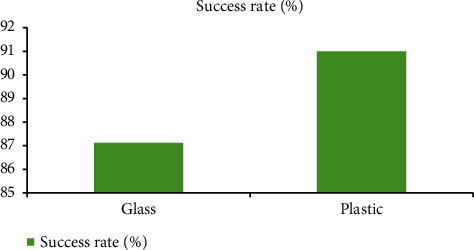
Success rate of grasping objects using suction.

**Table 1:** Success rate of grasping objects using suction.

Category	Object configuration	Number of objects	Number of attempts	Number of successes (%)	Success rate (%)

Glass	VG	13	71	89.58	87.12
	NVG	10	46	83.33	

Plastic	VP	5	21	91.1	91
	NVP	7	31	91	

**Table 2:** Success rate of grasping objects using gripper.

Category	Object configuration	Number of objects	Number of attempts	Number of successes (%)	Success rate (%)

Glass	VG	6	26	85.2	87.78
	NVG	11	51	89.5	

Plastic	VP	5	26	77.21	69.86
	NVP	12	56	66.47	

**Table 3:** Success rate of object classifications.

Category	Object configuration	Number of objects	Number of attempts	Number of successes (%)	Success rate (%)

Glass	VG	7	31	99.9	86.4
	NVG	15	71	79.68	

Plastic	VP	8	36	86.82	87.1
	NVP	14	65	87.26	

**Table 4:** Accuracy and loss of train and test phase results.

S. No	Cycles	Test phase	Train phase

1	40	0.2	0.4
2	80	0.6	0.7
3	120	0.7	0.7
4	160	0.8	0.9
5	200	0.9	1.0

## Data Availability

The datasets used and/or analyzed during this study are available from the corresponding author on reasonable request.
